# Editorial: Sudden deafness

**DOI:** 10.3389/fneur.2024.1520018

**Published:** 2024-11-13

**Authors:** Jun Yang, Yupeng Liu, Lisheng Yu, Maoli Duan

**Affiliations:** ^1^Department of Otorhinolaryngology-Head and Neck Surgery, Xinhua Hospital, Shanghai Jiaotong University School of Medicine, Shanghai, China; ^2^Shanghai Jiaotong University School of Medicine Ear Institute, Shanghai, China; ^3^Shanghai Key Laboratory of Translational Medicine on Ear and Nose Diseases, Shanghai, China; ^4^Department of Otorhinolaryngology-Head and Neck Surgery, People's Hospital of Peking University, Beijing, China; ^5^Ear Nose and Throat Patient Area, Trauma and Reparative Medicine Theme, Karolinska University Hospital, Stockholm, Sweden; ^6^Division of Ear, Nose and Throat Diseases, Department of Clinical Science, Intervention and Technology, Karolinska Institutet, Stockholm, Sweden

**Keywords:** sudden deafness, sensorineural hearing loss, diagnosis, treatment, comorbidity

## Introduction

Sudden sensorineural hearing loss (SSNHL) is an otologic emergency characterized by a rapid onset of hearing loss, typically within 72 h, affecting three or more consecutive frequencies by 30 dB or more (1). The global incidence of SSNHL ranges from 5 to 400 per 100,000 individuals annually, with a rising trend worldwide (2). Although viral infections, autoimmune diseases, and vascular abnormalities are acknowledged as potential etiological factors, the pathophysiology of SSNHL remains unclear in the majority of cases (3). Current treatment strategies focus on the use of corticosteroids-either systemic or intratympanic-as the primary therapy to reduce inflammation and restore hearing (4). Additional treatments such as hyperbaric oxygen therapy, traditional Chinese medicine (TCM), and vasodilators have been explored but with inconsistent evidences. However, the effectiveness of alternative therapies continues to be the subject of ongoing debate. Accurately predicting hearing recovery is essential for patient counseling, as factors such as delayed treatment initiation, vestibular function impairment, and comorbid health conditions are associated with poorer prognoses. This Research Topic “*Sudden deafness*” consists of 16 original articles and two reviews. We summarized these articles within the following categories: overview of SSNHL in China, comorbidities and laboratory changes, special types of SSNHL, therapeutic regimen and prognostic factors. Further understanding of SSNHL through this Research Topic will benefit SSNHL patients and their families, thus decreasing the economic load for the society.

## Overview of SSNHL in china

Chen N. et al. provides a comprehensive overview of the contemporary clinical approaches to diagnosing and treating SSNHL in China. The study evaluated the factors influencing these practices, such as hospital grade and regional economic differences. The study highlighted the heterogeneity in SSNHL diagnosis and treatment in China. There is a need for more standardized practices and higher-quality RCT studies to ensure better outcomes. The widespread use of post-auricular injections and combination therapies in China is noted as a distinct practice, which may inform future international research and treatment guidelines.

## Comorbidities and laboratory changes

Certain comorbidities are recognized as risk factors for SSNHL, and specific laboratory findings may offer insights into the etiology of SSNHL. Xie, Karpeta, Tong et al. provided a review focuses on the etiological comorbidities and laboratory changes of SSNHL. They concluded that various etiological comorbidities have been associated with SSNHL, including cardiovascular diseases, metabolic diseases, autoimmune diseases, et al. They also pointed out that abnormal laboratory tests, including blood coagulation, endothelial dysfunction and inflammation, were reported in SSNHL patients. The review emphasizes the need for further research into the comorbidities and laboratory findings related to SSNHL to develop more effective and targeted treatments. Chen J. et al. investigates the causal relationship between thyroid function and SSNHL using Mendelian randomization (MR). The results suggest that genetically predicted elevated FT4 levels may reduce the risk of SSNHL, while no significant association was found between TSH levels and SSNHL. Zeng et al. developed and validated a predictive model for SSNHL. The model identified thrombin time (TT), red blood cell (RBC) count, and granulocyte-lymphocyte ratio (GLR) as key predictors. This prediction model could aid in early diagnosis and treatment decisions for SSNHL. Zhang J. et al. found that miRNAs may be closely related to SSNHL pathogenesis and could serve as potential biomarkers for early diagnosis and prognosis. do Amaral et al. explored the relationship between inflammatory markers, metabolic parameters, and hearing recovery in SSNHL patients. Key findings included a significant decrease in cytokines such as TNF-α and IFN-γ over time, which correlated with hearing improvement. Zhong et al. explored the association between SSNHL and stroke, particularly posterior circulation strokes. The study emphasizes the importance of early audiometric and vascular assessments in SSNHL patients to detect and prevent stroke, particularly in high-risk individuals. These studies collectively contribute to a better understanding of SSNHL by exploring its association with thyroid function, miRNA profiles, stroke risk, and inflammatory processes.

## Special types of SSNHL

Special populations and unique categories of SSNHL garnered insufficient attention yet, and clinical research in this domain remains limited. In contrast to common forms of SSNHL, these specific cases demonstrated differences in both etiology and prognosis. Liu et al. investigated the clinical features and prognosis of SSNHL in single-sided deafness (SSD) patients. The SSD group had poorer hearing recovery outcomes and lower hearing gains after treatment. Patients with SSNHL in the sole hearing ear face significant challenges in recovery, emphasizing the need for optimized treatment strategies. He et al. focused on bilateral sudden sensorineural hearing loss (BSSNHL). The study found that patients with BSSNHL tend to have more severe hearing loss and worse prognosis than those with unilateral SSNHL. The overall treatment efficacy was 32%, with those having profound hearing loss showing worse outcomes. Wang et al. also evaluates the clinical characteristics and prognosis of BSSNHL compared with unilateral SSNHL. BSSNHL patients showed poorer hearing recovery and more severe symptoms. Prognostic factors included the audiogram curve type, with sloping-type audiograms being linked to worse outcomes. Li et al. conducted a bi-center retrospective study analyzed 145 pediatric SSNHL patients to identify factors influencing prognosis. Children with ascending and flat form of audiogram configurations had better recovery, while descending ones were associated with worse outcomes. The study also found that higher platelet-to-lymphocyte ratios (PLR) and lower lymphocyte counts were related to worse initial hearing loss, highlighting the role of systemic inflammation in pediatric SSNHL. Further clinical research is imperative to investigate the underlying mechanisms and treatment strategies for these cases to enhance patient outcomes.

## Therapeutic regimen

The established effective treatment for SSNHL involves the systemic administration and intratympanic injection of corticosteroids. However, the efficacy of alternative administration methods, including post auricular injections, repetitive systemic corticosteroid administration, and TCM, requires further investigation. Xie, Karpeta, Liu et al. investigated whether adding intratympanic or post auricular subperiosteal corticosteroid injections to systemic corticosteroid treatment improves hearing recovery in patients with SSNHL. The study found no significant difference in hearing recovery between the groups, indicating that local corticosteroid injections do not significantly improve outcomes when added to systemic corticosteroids. Yamamoto et al. compared patients who received repetitive treatment with those who only received one round of therapy. Although the final hearing outcomes did not differ significantly between the groups, early and sufficient corticosteroid dosing was found to be crucial for better hearing recovery. Although repetitive corticosteroid therapy may play a supplementary role, the study emphasizes the importance of early, aggressive treatment to improve outcomes. Zhao et al. used network pharmacology and molecular docking techniques to investigate the molecular mechanisms by which the TCM Erlongjiaonang (ELJN) acts in the treatment of SSNHL. The study suggests that ELJN may reduce inflammation and improve inner ear blood circulation, providing a molecular basis for its effectiveness in treating SSNHL.

## Prognostic factors

The prognosis of SSNHL is generally influenced by factors including the patient's age, the severity of the initial hearing loss, the timing of treatment initiation, and the presence of vestibular function impairment. Prior research examining the correlation between vestibular function impairment and the prognosis of SSNHL remains limited. Chen L. et al. explored the prognosis of patients with SNHL who also had inner ear malformations involving the lateral semicircular canal (LSCC). Compared with patients without LSCC malformation, the recovery outcomes were poorer, with only 40% of patients LSCC malformation showing hearing improvement. The study suggests that LSCC malformation is a risk factor for poor prognosis in SSNHL. Shen et al. examined the functional status of the vestibular otolith and conductive pathways in unilateral SSNHL patients using vestibular evoked myogenic potentials (VEMPs). The study found that patients with normal VEMPs had better hearing recovery than those with abnormalities. The study highlights the importance of evaluating both the otolith and vestibular nerve pathways to predict hearing outcomes. Yang et al. investigated the relationship between vestibular function and prognosis in patients with severe and profound SSNHL. The findings suggest that vestibular ischemia caused by corresponding vascular circulation disorder affect both the cochlea and posterior semicircular canal, may contribute to poor outcomes in these patients. These studies focus on the impact of vestibular function and inner ear abnormalities on the prognosis of patients with SSNHL, emphasizing the need for comprehensive vestibular assessment to better understand and predict recovery outcomes. In addition, cardiovascular disease, diabetes, hypercholesterolemia and hypertension have been found to be poor prognostic factors. However, randomized double blind placebo control study has not been investigated until now. Thus, it is difficult to say that these factors affect SSNHL's prognosis.

### Prospect

SSNHL, an otologic emergency, has a wide incidence globally, and its pathophysiology remains largely unknown. Therefore, future research in SSNHL should focus on uncovering the underlying pathophysiology. Advancements in molecular biology, genetics, and bioinformatics could provide deeper insights into the causes of SSNHL. Studies investigating biomarkers, including miRNAs, inflammatory markers, and genetic factors, could help identify high-risk individuals and improve early diagnosis. Additionally, more high-quality, randomized double blind placebo controlled trials are needed to validate the effectiveness of alternative therapies like TCM and novel drug combinations.
